# Sivelestat sodium attenuates acute lung injury by inhibiting JNK/NF-κB and activating Nrf2/HO-1 signaling pathways

**DOI:** 10.17305/bb.2022.8549

**Published:** 2023-05-01

**Authors:** Hong Zhang, Jun Zeng, Jiankang Li, Huankai Gong, Meiling Chen, Quan Li, Shengxing Liu, Shanjun Luo, Huanxiang Dong, Yingke Xu, Huanling Duan, Ling Huang, Chuanzhu Lv

**Affiliations:** 1 Emergency and Trauma College, Hainan Medical University, Haikou, Hainan, China; 2 Key Laboratory of Emergency and Trauma of Ministry of Education, Hainan Medical University, Haikou, Hainan, China; 3 Emergency Medicine Center, Sichuan Provincial People’s Hospital, University of Electronic Science and Technology of China, Chengdu, Sichuan, China; 4 Department of Emergency, State Key Laboratory of Complex Severe and Rare Diseases, Peking Union Medical College Hospital, Chinese Academy of Medical Science and Peking Union Medical College, Beijing, China; 5 Department of Orthopedics, The Second Affiliated Hospital of Hainan Medical University, Haikou, Hainan, China; 6 Intensive Care Unit, Chenzhou Third People’s Hospital, Chenzhou, Hunan, China; 7 Hainan Pharmacopoeia Review Center of Hainan Provincial Drug Administration, Haikou, Hainan, China; 8 State Key Laboratory of Trauma, Burns and Combined Injury, Department of Wound Infection and Drug, Daping Hospital, Army Medical University, Chongqing, China; 9 Research Center for Drug Safety Evaluation of Hainan Province, Hainan Medical University, Haikou, Hainan, China; 10 Research Unit of Island Emergency Medicine, Chinese Academy of Medical Sciences (No. 2019RU013), Hainan Medical University, Haikou, Hainan, China

**Keywords:** Sivelestat sodium (SIV), acute lung injury (ALI), inflammation, oxidative stress, JNK/NF-κB, Nrf2/HO-1

## Abstract

Sivelestat sodium (SIV), a neutrophil elastase inhibitor, is mainly used for the clinical treatment of acute respiratory distress syndrome (ARDS) or acute lung injury (ALI). However, studies investigating the effects of SIV treatment of ALI are limited. Therefore, this study investigated the potential molecular mechanism of the protective effects of SIV against ALI. Human pulmonary microvascular endothelial cells (HPMECs) were stimulated with tumor necrosis factor α (TNF-α), and male Sprague–Dawley rats were intratracheally injected with *Klebsiella pneumoniae* (KP) and treated with SIV, ML385, and anisomycin (ANI) to mimic the pathogenetic process of ALI in vitro and in vivo, respectively. The levels of inflammatory cytokines and indicators of oxidative stress were assessed in vitro and in vivo. The wet/dry (W/D) ratio of lung tissues, histopathological changes, inflammatory cells levels in bronchoalveolar lavage fluid (BALF), and survival rates of rats were analyzed. The JNK/NF-κB (p65) and Nrf2/HO-1 levels in the HPMECs and lung tissues were analyzed by western blot and immunofluorescence analyses. Administration of SIV reduced the inflammatory factors levels, intracellular reactive oxygen species (ROS) production, and malondialdehyde (MDA) levels and increased the levels of superoxide dismutase (SOD) and glutathione peroxidase (GSH-Px) in lung tissues. Meanwhile, SIV alleviated pathological injuries, decreased the W/D ratio, and inflammatory cell infiltration in lung tissue. In addition, SIV also inhibited the activation of JNK/NF-κB signaling pathway, promoted nuclear translocation of Nrf2, and upregulated the expression of heme oxygenase 1 (HO-1). However, ANI or ML385 significantly reversed these changes. SIV effectively attenuated the inflammatory response and oxidative stress. Its potential molecular mechanism was related to the JNK/NF-κB activation and Nrf2/HO-1 signaling pathway inhibition. This further deepened the understanding of the protective effects of SIV against ALI.

## Introduction

Acute lung injury (ALI) is an inflammatory disease characterized by acute lung tissue damage and pulmonary dysfunction, which are caused by infectious or aseptic injury [1]. Its main features include extensive destruction of the pulmonary alveolar epithelial cells and capillary endothelium, which impair the blood–air barrier and causing large amounts of fluid to accumulate in the alveoli and interstitial spaces of the lungs [2, 3]. Clinically, ALI can cause severe respiratory distress, progressive hypoxemia, acute respiratory distress syndrome (ARDS), and even death. Despite the significant advances in its treatment methods and organ preservation measures, ALI mortality is still as high, ranging from 30% to 40% in most cases [4]. Therefore, discovering more efficient approaches for the treatment of ALI is necessary.

The pathophysiology processes and molecular mechanisms of ALI have not been clearly elucidated. However, accumulating evidence has shown that inflammatory stimuli and oxidative stress damage are essential pathological mechanisms, exacerbating its severity [5–7]. In ALI, a large number of immune cells, such as neutrophils, macrophages, lymphocytes, platelets, etc., are recruited and overactivated in the lungs, increasing the proinflammatory cytokine levels, including interleukin β (IL-β), IL-8, and tumor necrosis factor α (TNF-α), which induce cytokine storm. Therefore, the pathogens and normal cells in the body are indiscriminately attacked, causing systemic inflammation and multiple organ failure [8, 9]. In addition, oxidative stress, another important factor, can lead to ALI development [10]. The oxidative and antioxidant systems of the body are in dynamic balance in the absence of external cellular or tissue-stimulating factors, ensuring low reactive oxygen species (ROS) levels, which is essential for maintaining normal cellular physiological processes [11]. However, the production of ROS markedly increases during ALI, while the endogenous antioxidant system is inhibited, thereby weakening the ROS-scavenging ability. That leads to damaging the alveolar epithelium and vascular endothelial cells, impairing the blood–air barrier, and aggravating pulmonary edema [12]. Therefore, reducing the levels of inflammatory factors and ROS might be an important therapeutic option to treat ALI.

Nuclear factor κB (NF-κB), which regulates the expression of many inflammation-related genes in an organism, is involved in cellular responses to various stimuli. Upon stimulation by external stimuli, the phosphorylation of NF-κB and its rapid nuclear translocation induces the release of proinflammatory factors [13]. Moreover, the c-Jun N-terminal kinase (JNK) signaling pathway is also involved in several pathophysiological responses, including cellular proliferation, apoptosis, and stress. Studies have shown a close correlation between the JNK signaling pathway and the inflammatory response [14, 15]. Increasing evidence suggested that inhibition of the JNK signaling pathway might attenuate ALI by reducing the production of inflammatory mediators [16, 17].

Nuclear factor erythroid 2-related factor 2 (Nrf2) can critically regulate the endogenous antioxidant pathway, and its activation can reduce oxidative stress-induced damage [18]. Under normal circumstances, Nrf2 remains in the cytoplasm, while oxidative stress can induce its rapid translocation into the nucleus, where it regulates the expression of different detoxifying and antioxidant substances, such as superoxide dismutase (SOD), glutathione peroxidase (GSH-Px), heme oxygenase 1 (HO-1), etc., in order to remove the superfluous ROS [19–21]. Consequently, inhibition of JNK and NF-κB and activation of Nrf2 signaling pathways might be an effective approach to treat ALI.

Sivelestat sodium (SIV), a highly specific neutrophil elastase (NE) inhibitor, has been clinically used to treat ALI by inhibiting the activity of NE [22] and was marketed rapidly in China during the novel coronavirus disease 19 (COVID-19) epidemic in 2020. Clinical studies showed that SIV could improve oxygenation indices, alleviate multi-organ dysfunction, and reduce the duration of mechanical ventilation, which eventually led to the improvement of patients’ survival [23]. Animal experimental studies showed that treatment with SIV could reduce the development of malaria-associated ALI/ARDS and significantly improve the survival rate of mice [24]. In the cecal ligation and perforation-induced sepsis rat models, the SIV treatment inhibited the NE activity, restored the mean arterial pressure and glomerular filtration rate of rats, reduced the release of proinflammatory factors, and improved the survival rate of rats [25]. Currently, the known pharmacological effect of SIV is the inhibition of NE. However, studies exploring the other potential of SIV for the treatment of ALI are limited.

The current study hypothesized that, in addition to inhibiting NE, SIV had the potential in the treatment of ALI. In order to test this hypothesis, this study investigated the efficacy of SIV in ALI treatment in vitro and in vivo and explored its effects on Nrf2/HO-1 and JNK/NF-κB signaling pathways.

## Materials and methods

### Materials and reagents

Injectable SIV was gifted by Shanghai Huilun Jiangsu Pharmaceutical Co., Ltd. (Jiangsu, China). Recombinant human TNF-α was purchased from PeproTech Company (New Jersey, USA). Cell Counting Kit 8 (CCK-8) and ROS Assay Kit were purchased from Biosharp Co., Ltd. (Hefei, China). The enzyme-linked immunosorbent assay (ELISA) kits for IL-1β, IL-8, and TNF-α were obtained from Shanghai FANKEL Industrial Co., Ltd. (Shanghai, China). The GSH-Px, malondialdehyde (MDA), and SOD assay kits were obtained from Nanjing Jiancheng Bioengineering Institute (Nanjing, China). ML385 (HY-100523) and anisomycin (ANI) (HY-18982) were purchased from Med Chem Express (New Jersey, USA). Eastep^®^ Super Total RNA Extraction Kit (LS1040) was obtained from Promega Corporation (Madison, Wisconsin, USA). Hifair^®^ &II 1st Strand cDNA Synthesis SuperMix for qPCR (gDNA digester plus) (11123ES60) was purchased from Yeasen Biotechnology Science and Technology Ltd. (Shanghai, China). Fetal bovine serum (FBS) and penicillin–streptomycin–amphotericin B (PSAB) were obtained from Procell Life Science & Technology Co., Ltd.

### Cell line and culture

Human pulmonary microvascular endothelial cells (HPMECs) were provided by Qingqi (Shanghai) Biotechnology Development Co., Ltd. (Shanghai, China) and cultured in a complete medium containing 90% Dulbecco’s Modified Eagle Medium (DMEM) (Gibco; ThermoFisher Scientific, Inc.), 10% FBS, and 1% PSAB. The cell culture was grown in a 5% CO_2_ incubator at 37 ^∘^C.

### Cell groups and treatments

First, the cell cultures were divided into different groups as follows: Control (Ctrl), TNF-α, TNF-α + SIV (50 µg/mL), and TNF-α + SIV (100 µg/mL) groups. When the HPMECs confluency reached 70% in the 6-well plate, the culture medium was replaced. Then, the Ctrl group cells were cultured for 24 h without any treatment. The TNF-α group cells were treated with 0.2 µg/mL TNF-α for 24 h, while the SIV (50, 100 µg/mL) group cells were first treated with SIV for 2 h, followed by treatment with TNF-α (0.2 µg/mL) and then co-cultured for 24 h. After culturing the cells for 24 h, they were harvested and used subsequently in the experiments.

Next, the HPMECs were co-treated with SIV (100 µg/mL), ANI, or ML385, and TNF-α for 24 h. The cells were grouped as follows: Ctrl, TNF-α, TNF-α + SIV (100 µg/mL), TNF-α + SIV (100 µg/mL) + ML385, and TNF-α + SIV (100 µg/mL) + ANI. After culturing the cells for 24 h, they were harvested and used subsequently in the experiments.

### CCK-8 assay

The density of HPMECs was adjusted to 1 × 10^4^ cells/mL and added (100 µL) to each well of the 96-well culture plate. After culturing the cells for 12 h in an incubator (5% CO_2_ and 37 ^∘^C), they were treated with SIV, TNF-α, ML385, and ANI for 24 h. The CCK-8 kit was used to measure the cellular viability according to the manufacturer’s instructions.

### Intracellular ROS detection

A 2 mL of HPMECs suspension (cell density of 1 × 10^4^ cells/mL) was added to the confocal small dishes. After cell adherence, the HPMECs were treated with SIV, TNF-α, and ML385 for 24 h. Then, the ROS levels were measured using an ROS assay kit. ROS fluorescence intensity was measured using ImageJ software.

### Animals and experimental design

Hunan SJA Laboratory Animal Co., Ltd. provided the male Sprague–Dawley rats (weighing 180–220 g and aged 12–14 weeks) (production license No. SCXK [Hunan] 2019–0014). *Klebsiella pneumonia* (KP) strains, purchased from National Institutes for Food and Drug Control, were cultured in Luria–Bertani (LB) agar medium for 12 h in a bacteria-specific incubator. The bacterial culture was collected and resuspended in normal saline. The bacterial concentrations were measured using Michaelis turbidimetric method and adjusted to 1.2 × 10^10^ CFU/mL. The rats were anesthetized by the intraperitoneal injection of 3% pentobarbital sodium (prediluted with sterile normal saline) at the dose of 50 mg/kg. Then, the bacterial suspension (0.2 mL) was injected into their trachea [26].

The rats were divided randomly into different groups as follows: Ctrl, ALI, ALI + SIV (50 mg/kg), ALI + SIV (100 mg/kg), ALI + SIV (100 mg/kg) + ANI, and ALI + SIV (100 mg/kg) + ML385 groups. The Ctrl group rats were kept reared without any special treatment. The ALI + SIV (50 mg/kg) group and ALI + SIV (100 mg/kg) group rats were intraperitoneally injected with SIV (50 and 100 mg/kg, respectively) [25, 27], for 6 days 12 h after injecting bacterial suspension. The ALI + SIV (100 mg/kg) + ANI group and ALI + SIV (100 mg/kg) + ML385 group rats were first intraperitoneally injected with ANI (1 mg/kg) or ML385 (30 mg/kg) for 3 days, followed by intratracheal injection of the bacterial suspension. After 12 h, SIV (100 mg/kg) was intraperitoneally injected for 6 days. Twelve hours after the last dose, bronchoalveolar lavage fluid (BALF), vein blood, and lung tissue samples were collected from each group of rats under anesthesia.

### Hematoxylin eosin staining

The rat lung tissues were fixed in paraformaldehyde (4%) and paraffinized. The paraffinized tissue sections were sliced into sections (3-µm-thick) and stained with hematoxylin eosin (H&E). Four random areas in each tissue section were examined using a fluorescence microscope (DM2500, Leica, Wetzlar, Germany). The lung injury scores were calculated according to the severity of structural damage to lung tissue, including alveolar hemorrhage, congestion, alveolar wall thickening, and neutrophil infiltration. The severity of lung injury was assessed by a grading scale (0–4): 0 ═ no injury; 1 ═ 1%–25% injury of the field; 2 ═ 26%–50% injury of the field; 3 ═ 51%–75% injury of the field; and 4 ═ diffused injury [3].

### BALF analysis

After the rats were anesthetized, their right main bronchus was isolated and ligated. Their left lung was flushed three times with 2 mL pre-cooled PBS (4 ^∘^C) to collect BALF. Then, the collected sample was centrifuged (500 *g*, 4 ^∘^C, 10 min), and 900 µL red cell lysis solution (Biosharp Biotechnology Co., LTD, Hefei, China) and 100 µL of pre-cooled PBS (4 ^∘^C) for 2 min were added to resuspend the precipitated cells. The red blood cells were then removed from BALF by centrifugation (500 *g*, 4 ^∘^C, 5 min). Next, PBS was added to resuspend the remaining cells, and the total cell number was calculated. Wright-Giemsa Staining Solution (Beyotime Biotechnology, Shanghai China) was used to stain the cells in order to calculate the percentage of neutrophils among at least 200 cells in each slide.

### Detection of oxidative stress indicators

An appropriate amount of rat lung tissue was taken and homogenized. Then, the respective kits for GSH-Px, SOD, and MDA were used to measure their levels as described in their respective manufacturer’s protocols.

### Quantitative RT-PCR

Total RNA extraction was performed using the Eastep^®^ Super Total RNA Extraction Kit, followed by reverse transcription (cDNA synthesis) with the Hifair^®^ II 1st Strand cDNA Synthesis SuperMix for qPCR (gDNA digester plus) using a cDNA synthesis system (Applied Biosystems, Thermo Fisher Scientific, Inc., USA). Then, qRT-PCR was performed on the CFX96 Touch Real-Time PCR Detection System (Bio-Rad, Hercules, CA, USA) using a Hieff^^®^^ qPCR SYBR Green Master Mix (Low Rox Plus). The primer sequences used for qRT-PCR were as follows: TNF-α F: 5’-AGCCCTGGTATGAGCCCATCTAT-3’; TNF-α R: 5’-TCCCAAAGTAGACCTGCCCAGAC-3’; IL-1β F: 5’-GCCAGTGAAATGATGGCTTATT-3’; IL-1β R: 5’-AGGAGCACTTCATCTGTTTAGG-3’; IL-8 F: 5’-AACTGAGAGTGATTGAGAGTGG-3’; IL-8 R: 5’-ATGAATTCTCAGCCCTCTTCAA-3’.

### ELISA

After collecting the blood samples, they were immediately centrifuged, followed by supernatant (serum) collection, and stored in a −80 ^∘^C refrigerator. The detection of inflammatory factors (IL-1β, IL-8, TNF-α) levels in serum was performed using their respective kits as described in their respective manufacturer’s protocols.

### Immunofluorescence staining

The fixation of HPMECs was performed using 4% paraformaldehyde followed by permeabilization using 1% Triton X-100 (Biosharp, China, BS084), followed by blocking for 10 min with quick blocking buffer (Beyotime, China, P0260) at room temperature. The paraffinized lung tissue sections were dewaxed, and antigen repair was performed, followed by blocking. Then, cells and lung tissues were incubated with Nrf2 (Proteintech, China, #16396-1-AP, 1:300) and p-p65 (CST, USA, #3033, 1:1600) primary antibodies for 10–16 h at 4 ^∘^C. After washing the excess primary antibodies the next day three times with pre-cooled PBS for 10 min, the tissue samples were incubated with the respective fluorescent CoraLite594-conjugated Goat Anti-Rabbit IgG (H+L) (Proteintech, China, #SA00013-4, 1:300) secondary antibodies. The mixture was then stained with an antifade mounting medium for fluorescence (with DAPI) (Biosharp, China, BL739A). The cells and tissues were then observed and photographed under laser confocal microscopy.

### Western blot

The total protein extraction from the cells and lung tissue was performed using the RIPA Lysis Buffer (Biosharp, China, BL504A). The extraction of both the nuclear and cytoplasmic proteins was performed using a cytoplasmic and nuclear protein extraction kit (Biosharp, BL670A, China). Electrophoresis was performed to separate the proteins using 8% sodium dodecyl sulfate–polyacrylamide gel electrophoresis (SDS-PAGE), followed by transferring them to polyvinylidene fluoride (PVDF) membranes. The blocking of membranes was performed using 5% skim milk powder or 5% bovine serum albumin (BSA) for 1.5 h at 37 ^∘^C. The membranes were then incubated for 10–16 h at 4 ^∘^C with primary antibodies: anti-p-JNK (Abcam, USA, ab76572, #1:3000), anti-JNK (Abcam, USA, ab179461, #1:1000), anti-p-p65 (CST, USA, #3033, 1:1000), anti-p65 (CST, USA, #8242, 1:500), anti-HO-1 (Abcam, USA, ab68477, 1:7000), anti-Nrf2 (GeneTex, USA, GTX103322, 1:500), anti-Lamin B1 (Proteintech, China, #12987-1-AP, 1:2000), and anti-GAPDH (Bioss, China, bs-2188R, 1:2000). After washing off the unbound primary antibodies, from the membranes, they were then incubated with the respective secondary antibodies (HRP-labeled goat anti-rabbit) (Biosharp, China, BL003A) at room temperature for 1.5 h. Finally, the membranes were developed using an ECL detection kit (Enhanced Chemiluminescence, Biosharp, China, BL520A). Image J software was applied to analyze the grayscale values of bands.

### Ethical statement

All the animal experiments were performed as per the guidelines of the Ethics Committee of Hainan Medical College (NO: HYLL-2021-159) and the National Institutes of Health’s Guide for the Care and Use of Laboratory Animals.

### Statistical analysis

All the experimental data were analyzed using GraphPad Prism 9.0 and expressed as mean ± standard deviation (SD). Tukey’s post-hoc test and one-way analysis of variance (ANOVA) were used to compare multiple groups, while Student’s t-test was used for comparing two groups. The survival rates were analyzed using Log-rank (Mantel-Cox) test and Kaplan–Meier survival curve analysis. Statistical significance was defined as a *P* value of < 0.05.

## Results

### SIV alleviated the destruction of rats’ lung tissues and increased their survival in vivo

**Figure 1.**
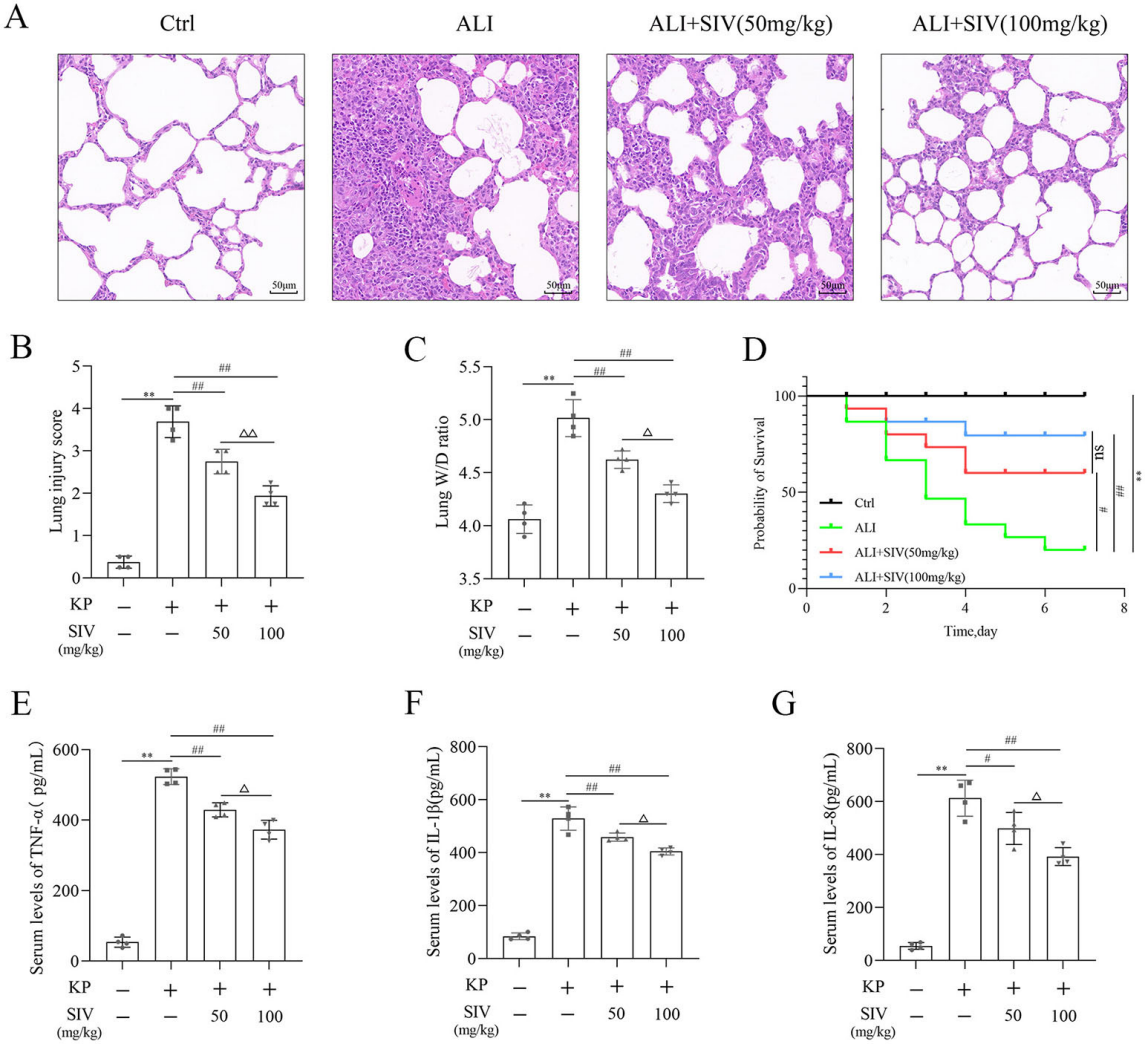
**SIV alleviated damage to the lung tissues and increased rat survival in vivo.** (A) Hematoxylin eosin staining of lung tissues (magnification, × 200); (B) Lung injury scores in different groups; (C) W/D ratio in lung tissues in different groups; (D) Survival plots for all the experimental rats (*n* ═ 15 rats per group). Inflammatory cytokines levels in serum (E) TNF-α, (F) IL-1β, and (G) IL-8 were determined using their respective ELISA kits in different groups. *P < 0.05, **P < 0.01; ^#^*P* < 0.05, ^##^*P* < 0.01; Δ*P* < 0.05, ΔΔ*P* < 0.01**.** SIV: Sivelestat sodium; ALI: Acute lung injury; KP: *Klebsiella pneumoniae*. Ctrl: Control; TNF-α: Tumor necrosis factor α; IL: Interleukin; W/D: Wet/dry.

First, the protective effects of SIV against KP-induced ALI were investigated. The H&E staining of rats’ lung tissues showed little or no damage in the Ctrl group, while in the ALI group showed severe damage to the lung tissues, including lung tissue proliferation, consolidation, numerous neutrophil infiltrations, and alveolar bleeding. However, the intraperitoneal injection of SIV (50 and 100 mg/kg) effectively reduced these lung lesions (Figure 1A and 1B). Meanwhile, the results also showed that the lung wet/dry (W/D) ratio, the serum levels of inflammatory cytokines, and the mortality rate of rats increased significantly in the ALI group and significantly decreased after the SIV administration (Figure 1C and 1G). These results indicated that SIV could alleviate lung tissue injury in KP-induced ALI in rats.

### SIV suppressed oxidative stress and inflammatory response and regulated related signaling pathways in vitro

First, the effects of SIV treatment for 24 h on the cell viability with or without TNF-α were analyzed. The SIV treatment at concentrations of 0–100 µg/mL had little impact on the viability of HPMECs. However, the concentrations above 100 µg/mL reduced the viability of the cells (Figure S1A). Therefore, 50 and 100 µg/mL SIV concentrations were selected for further analyses. Compared to the Ctrl group, the viability of HPMECs decreased in the TNF-α group, which increased significantly after the SIV administration (50 and 100 µg/mL) (Figure S1B). These results indicated that SIV could enhance cell viability in vitro.

**Figure 2.**
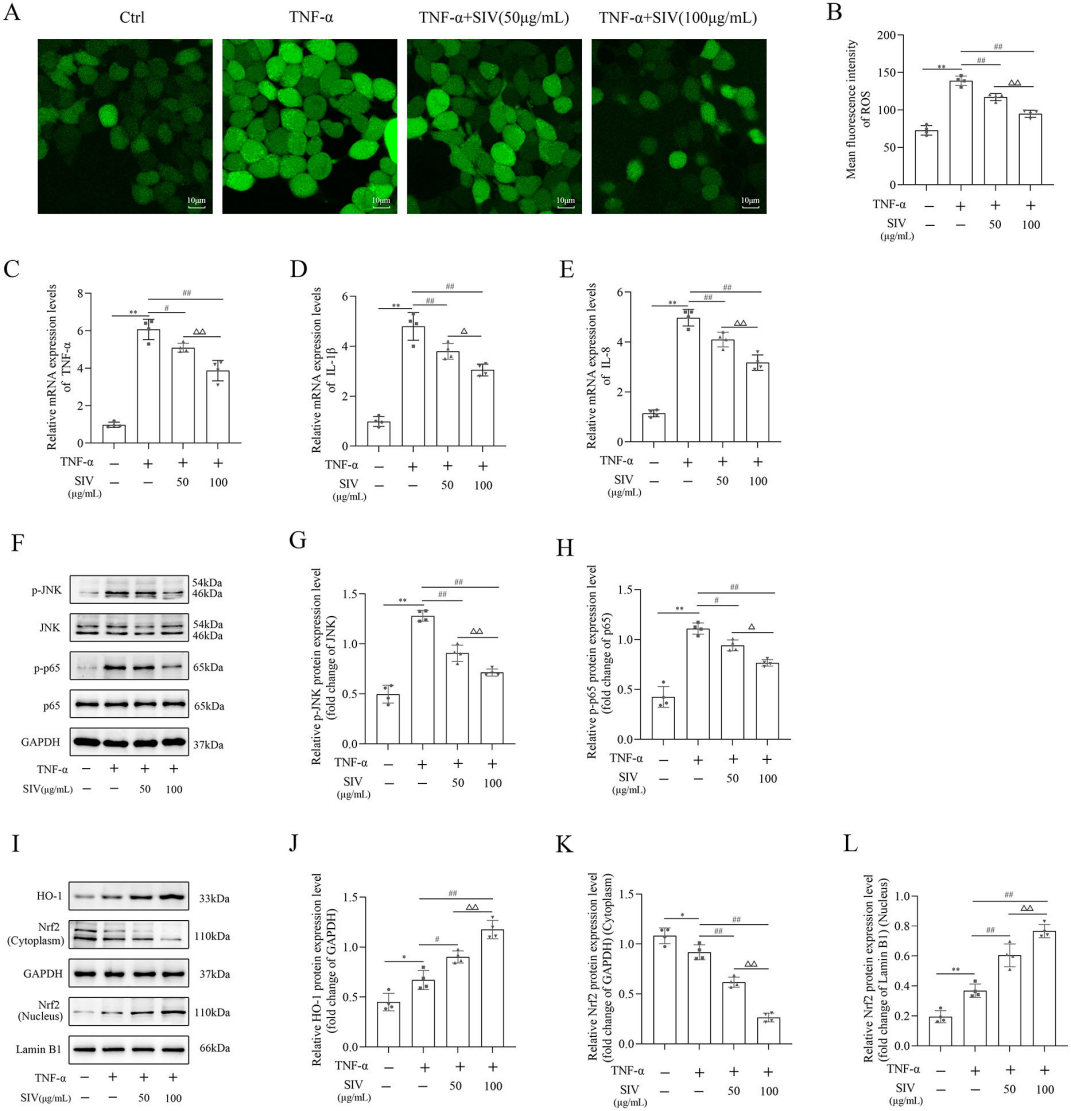
**SIV inhibited oxidative stress and inflammatory response and modulated JNK/NF-κB and Nrf2/HO-1 signaling pathways in vitro**. (A) and (B) ROS levels were detected in different groups. mRNA expression levels of (C) TNF-α, (D) IL-1β, and (E) IL-8 inflammatory cytokines were measured using RT-qPCR in different groups. (F)–(L) Expression levels of JNK/NF-κB and Nrf2/HO-1 signaling pathways-related proteins were analyzed using western blot in different groups. *P < 0.05, **P < 0.01; ^#^*P* < 0.05, ^##^*P* < 0.01; Δ*P* < 0.05, ΔΔ*P* < 0.01. ROS: Reactive oxygen species; Ctrl: Control; SIV: Sivelestat sodium; TNF-α: Tumor necrosis factor α; IL: Interleukin; JNK: c-Jun N-terminal kinase; NF-κB: Nuclear factor κB; Nrf2: Nuclear factor erythroid 2-related factor 2; HO-1: Heme oxygenase 1.

Next, the inflammatory and oxidative stress markers levels were detected to investigate if SIV had anti-inflammatory and antioxidant effects on the TNF-α-stimulated HPMECs. The results demonstrated that the ROS levels in the TNF-α-treated cells were noticeably higher, while the SIV treatment at 50 and 100 µg/mL markedly reduced the ROS production (Figure 2A and 2B). Additionally, TNF-α-stimulated HPMECs had significantly higher IL-1β, IL-8, and TNF-α levels than those in the Ctrl group, which gradually decreased after the SIV treatment (Figure 2C and 2E). Therefore, these findings suggested that SIV could reduce inflammatory cytokine levels and ROS production in vitro.

This study further explored the effects of SIV on JNK/NF-κB and Nrf2/HO-1 signaling pathways. In the TNF-α group, the p-JNK and p-p65 levels markedly increased. However, the SIV treatment (50 and 100 µg/mL) prevented the phosphorylation of JNK and NF-κB (Figure 2F and 2H). Furthermore, western blot analysis revealed that the SIV treatment accelerated the nuclear translocation of Nrf2 from the cytoplasm, therefore increasing the expression levels of HO-1 in the TNF-α-stimulated HPMECs (Figure 2I and 2L). Overall, these results indicated that SIV could limit oxidative stress and inflammatory response and modulate Nrf2/HO-1 and JNK/NF-κB signaling pathways in vitro.

### SIV alleviated oxidative stress and inflammatory response by Nrf2/HO-1 and JNK/NF-κB signaling pathways in vitro

**Figure 3.**
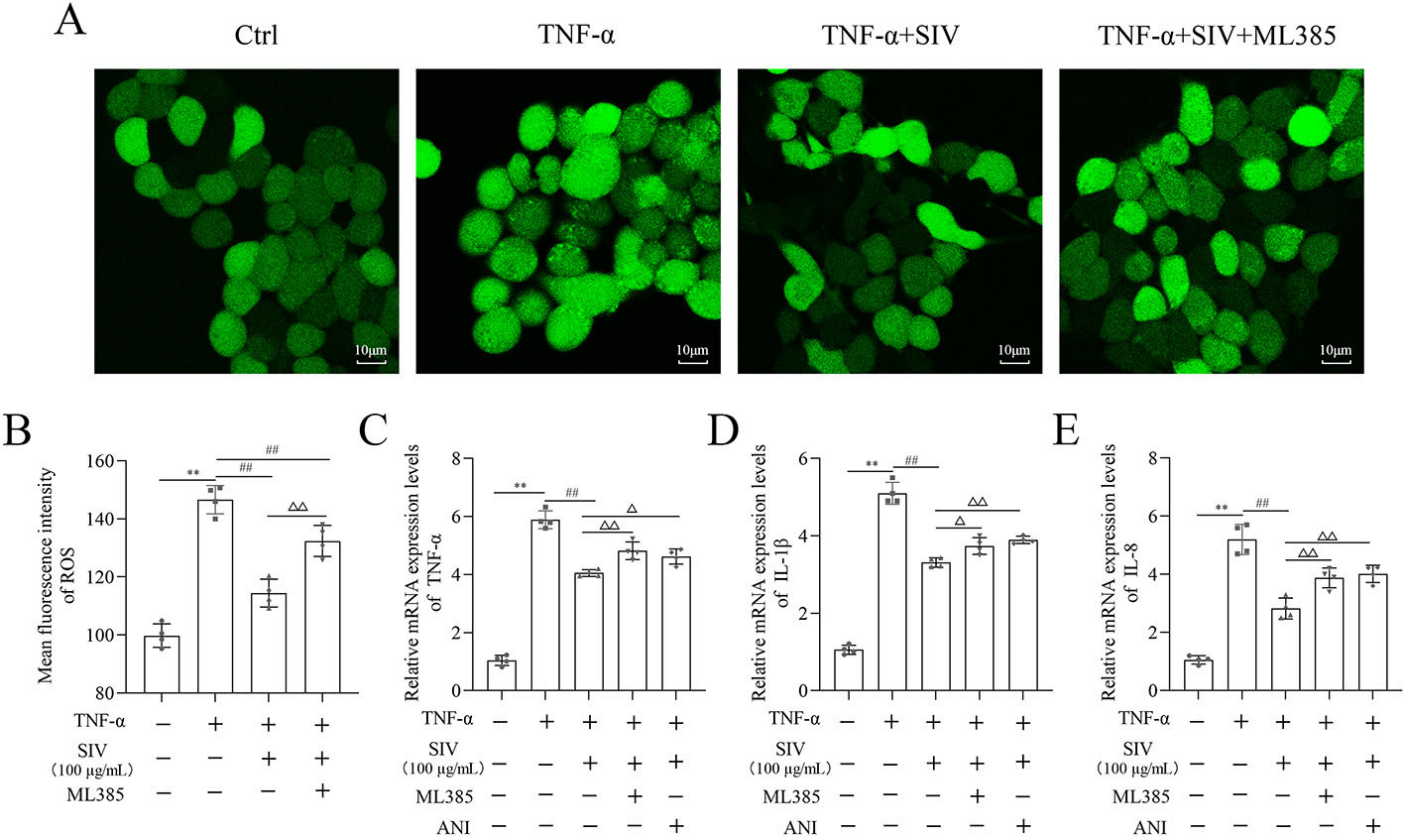
**Therapeutic effects of SIV alleviated oxidative stress and inflammatory response inhibited by ML385 or ANI in vitro.** (A) and (B) ROS levels were detected in different groups. mRNA expression levels of (C) TNF-α, (D) IL-1β, and (E) IL-8 inflammatory cytokines were measured using RT-qPCR in different groups. *P <0.05, **P <0.01; ^#^*P* <0.05, ^##^*P* <0.01; Δ*P* <0.05, ΔΔ*P* < 0.01. Ctrl: Control; ROS: Reactive oxygen species; SIV: Sivelestat sodium; ANI: Anisomycin; TNF-α: Tumor necrosis factor α; IL: Interleukin.

**Figure 4.**
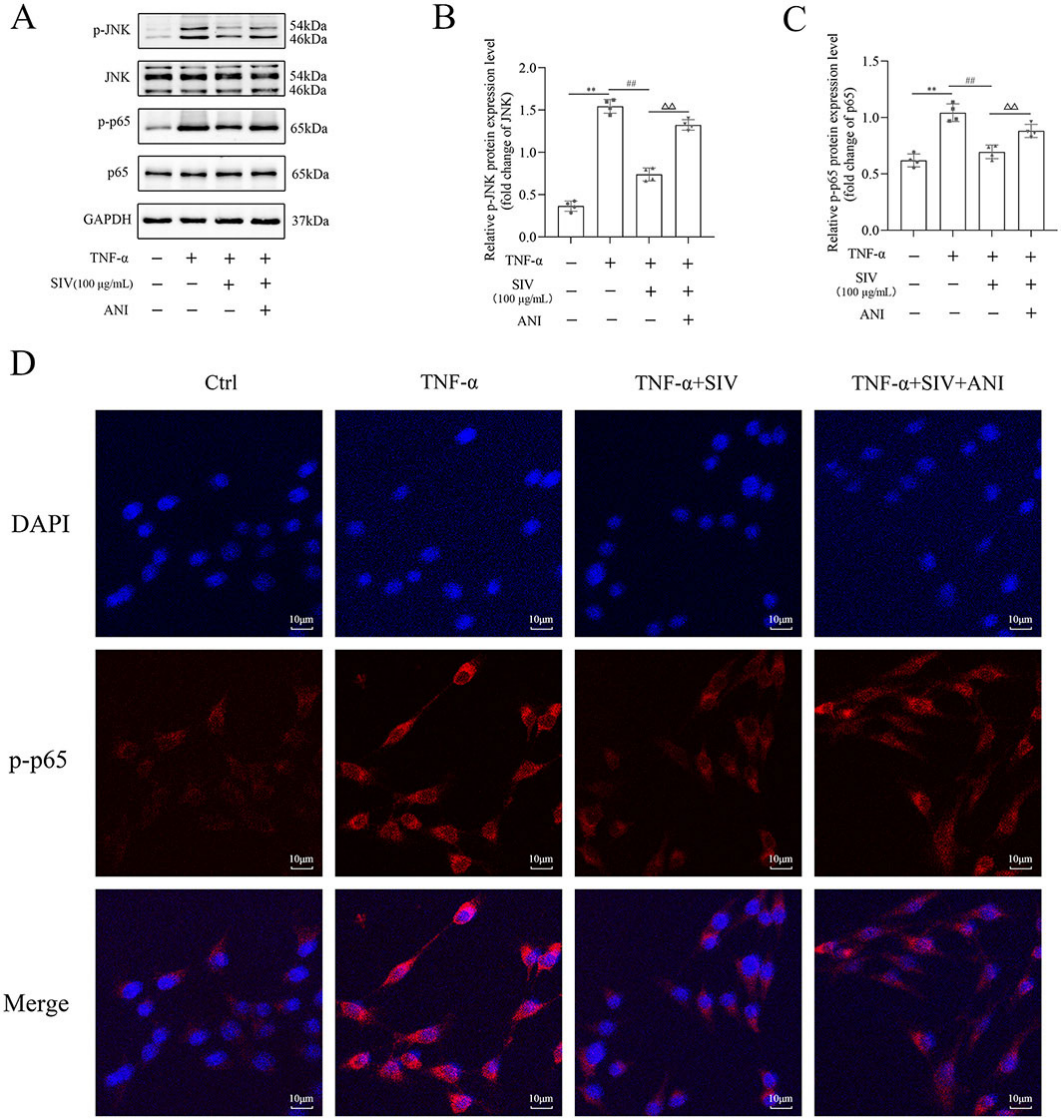
**ANI impaired SIV-suppressed activation of JNK/NF-κB signaling pathway in vitro.** Expression levels of (A)–(C) JNK/NF-κB signaling pathways-related proteins and relative quantification levels were analyzed using western blot in different groups. Immunofluorescence staining of (D) p-p65 were analyzed in different groups. *P <0.05, **P <0.01; ^#^*P* <0.05, ^##^*P* <0.01; Δ*P* <0.05, ΔΔ*P* < 0.01. Ctrl: Control; TNF-α: Tumor necrosis factor α; SIV: Sivelestat sodium; ANI: Anisomycin; JNK: c-Jun N-terminal kinase; NF-κB: Nuclear factor κB.

**Figure 5.**
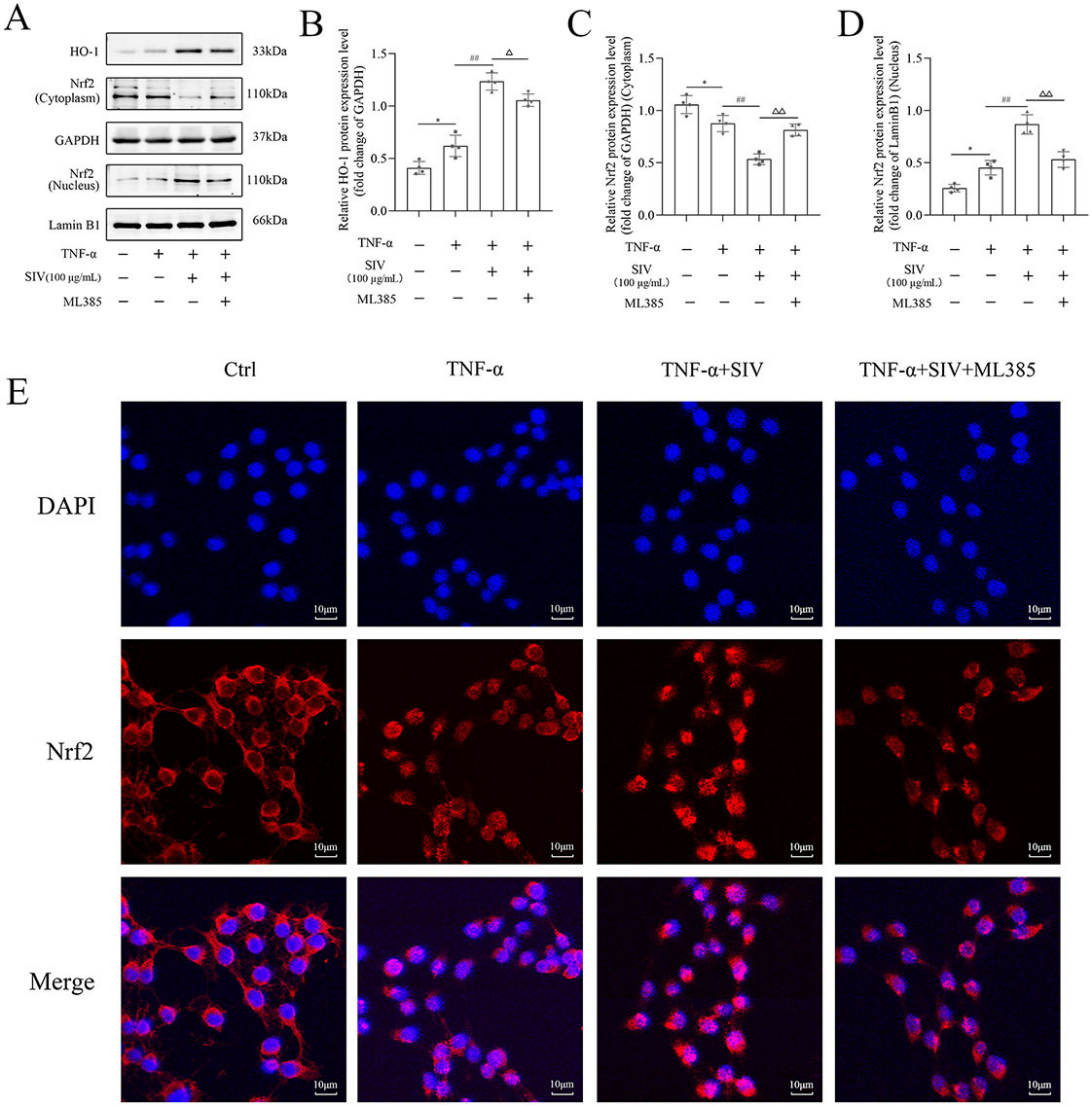
**ML385 impaired SIV-activated Nrf2/HO-1 signaling pathway in vitro.** Expression levels of (A)–(D) Nrf2/HO-1 signaling pathways-related proteins and relative quantification levels were analyzed using western blot in different groups. Immunofluorescence staining of (E) Nrf2 were analyzed in different groups. *P <0.05, **P <0.01; ^#^*P* <0.05, ^##^*P* <0.01; Δ*P* <0.05, ΔΔ*P* < 0.01. Ctrl: Control; TNF-α: Tumor necrosis factor α; SIV: Sivelestat sodium; ANI: Anisomycin; Nrf2: Nuclear factor erythroid 2-related factor 2; HO-1: Heme oxygenase 1.

Several experiments were performed in TNF-α-stimulated HPMECs treated with SIV (100 µg/mL), ML385, and ANI to investigate if SIV could inhibit the release of inflammatory cytokines by attenuating oxidative stress through the JNK/NF-κB and Nrf2/HO-1 signaling pathways. First, the results of CCK-8 showed that the SIV treatment attenuated the inhibitory effects of ML385 or ANI on cell viability (Figure S1C). The results further demonstrated that the administration of SIV decreased the ROS levels, while the addition of ML385 increased the expressed ROS levels to some extent (Figure 3A and 3B). In addition, in the TNF-α + SIV group, SIV also decreased the levels of inflammatory factors, while this inhibitory effect of SIV was weakened by ML385 or ANI (Figure 3C and 3E). Western blot revealed that SIV markedly reduced the phosphorylation levels of JNK and NF-κB compared to those in the TNF-α group; however, ANI reversed this inhibitory effect (Figure 4A and 4C). Immunofluorescence staining revealed that the SIV significantly decreased the TNF-α-induced production of p-p65 and inhibited its nuclear translocation; this effect was reversed by ANI (Figure 4D). In addition, western blot and cellular immunofluorescence analyses showed that the addition of ML385 inhibited the Nrf2’s nuclear translocation from the cytoplasm and reduced the expression levels of HO-1 (Figure 5A and 5E). Therefore, these results indicated that SIV could inhibit releasing the inflammatory cytokines by attenuating oxidative stress via JNK/NF-κB and Nrf2/HO-1 signaling pathways in vitro.

### ML385 or ANI inhibited the therapeutic effects of SIV on ALI in vivo

**Figure 6.**
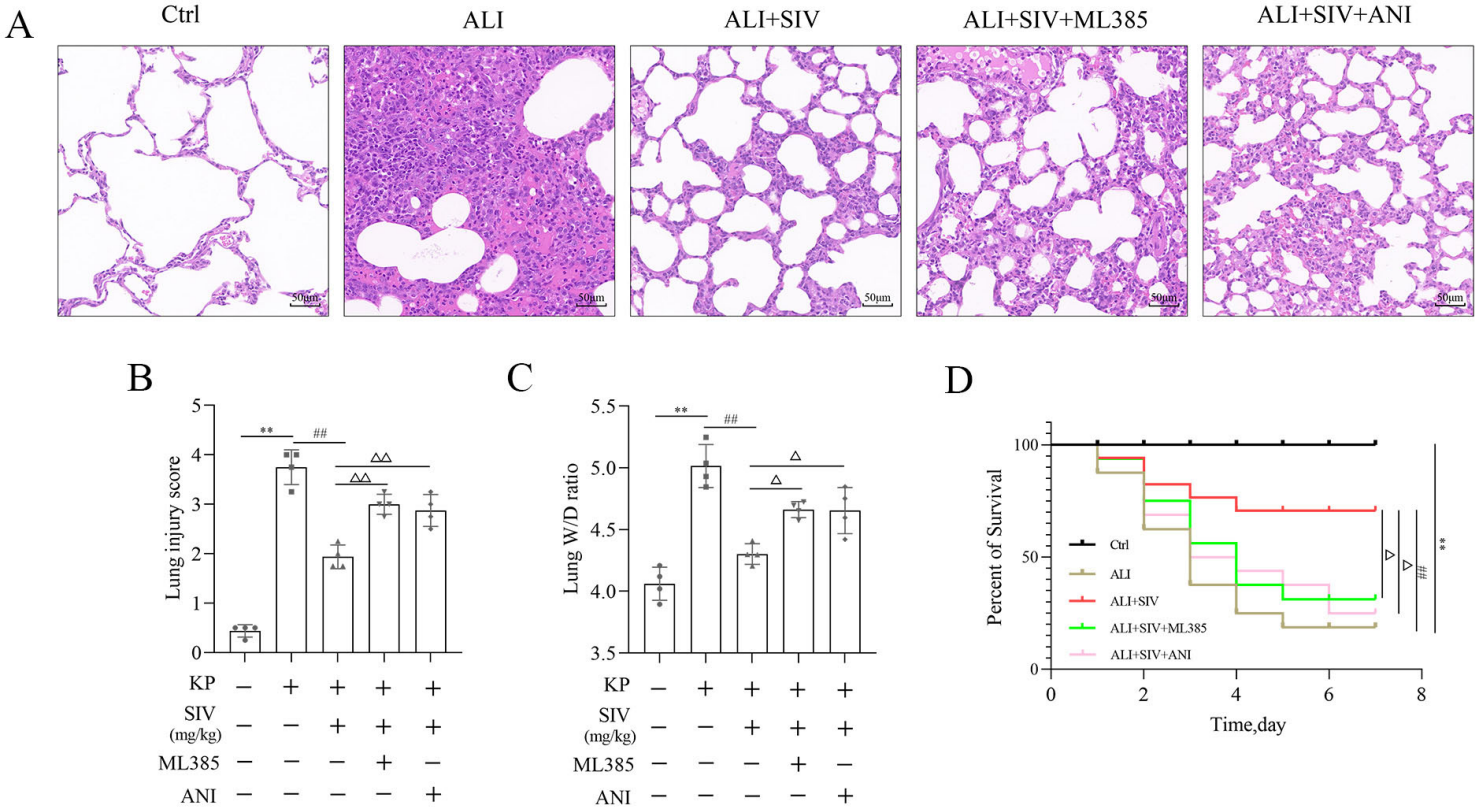
**Therapeutic effects of SIV on ALI were inhibited by ML385 or ANI in vivo.** (A) Hematoxylin eosin staining in lung tissues (magnification, ×200); (B) Lung injury score in different groups; (C) W/D ratio in lung tissues in different groups; (D) Survival plots for all the experimental rats (n ═ 15 rats per group). *P < 0.05, **P < 0.01; ^#^*P* < 0.05, ^##^*P* < 0.01; Δ*P* < 0.05, ΔΔ*P* < 0.01. Ctrl: Control; SIV: Sivelestat sodium; ALI: Acute lung injury; KP: *Klebsiella pneumoniae*; ANI: Anisomycin; W/D: Wet/dry.

The rats were treated with SIV (100 mg/kg), ML385, and ANI to further explore the therapeutic effects of SIV in KP-induced ALI rat models. The pathological changes in the lung tissues of ALI group rats included diffused proliferation, consolidation, numerous neutrophils infiltration, alveolar wall thickening, and alveolar hemorrhage. All these changes were alleviated in the ALI + SIV group. However, ML385 or ANI reversed the protective effects of SIV against pulmonary damage (Figure 6A). The lung injury score showed similar results (Figure 6B). In addition, evaluating the lung W/D ratio showed that the SIV treatment alleviated pulmonary edema compared to the ALI group; ML385 or ANI partly inhibited the SIV effects (Figure 6C). The survival plot analysis showed that SIV significantly improved the rat survival rate in KP-induced ALI, which decreased significantly by the ML385 or ANI administration (Figure 6D). These results showed that the ML385 or ANI administration could attenuate the protective effects of SIV on ALI rats.

### SIV alleviated oxidative damage and inflammation by inhibiting the JNK/NF-κB signaling pathway and activating the Nrf2/HO-1 signaling pathway in vivo

**Figure 7.**
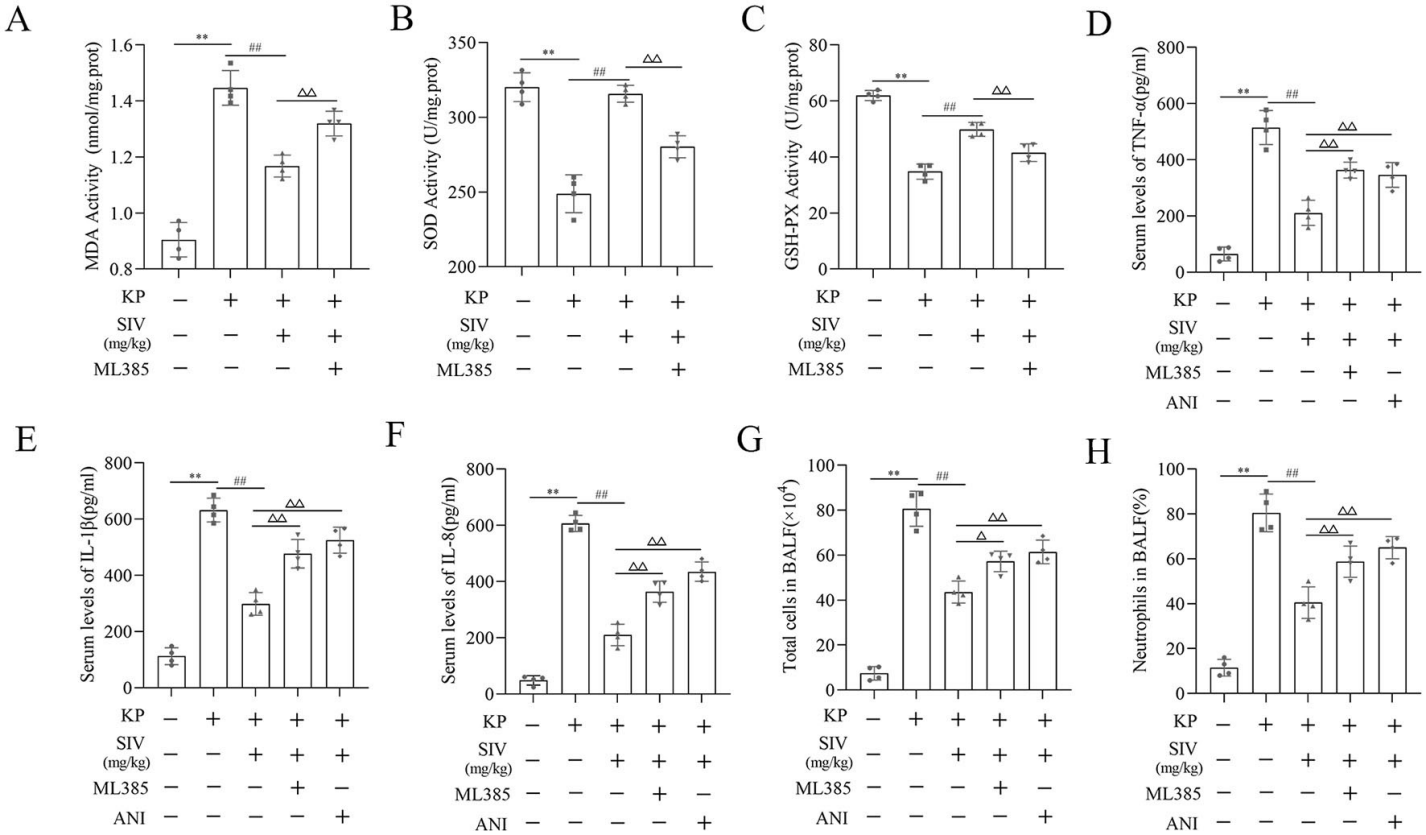
**Therapeutic effects of SIV alleviated oxidative stress-induced damage and inflammation inhibited by ML385 or ANI in vivo.** (A) MDA, (B) SOD, and (C) GSH-Px levels were determined in lung tissues in different groups. (D)–(F) Serum levels of inflammatory cytokines were determined using their respective ELISA kits in different groups. (G) Total cell counts and (H) percentage of neutrophils were determined in the BALF in different groups. *P <0.05, **P <0.01; ^#^*P* <0.05, ^##^*P* <0.01; Δ*P* <0.05, ΔΔ*P* <0.01. Ctrl: Control; SIV: Sivelestat sodium; ALI: Acute lung injury; KP: *Klebsiella pneumoniae*; ANI: Anisomycin; MDA: Malondialdehyde; SOD: Superoxide dismutase; GSH-Px: Glutathione peroxidase; BALF: Bronchoalveolar lavage fluid; TNF-α: Tumor necrosis factor α; IL: Interleukin.

**Figure 8.**
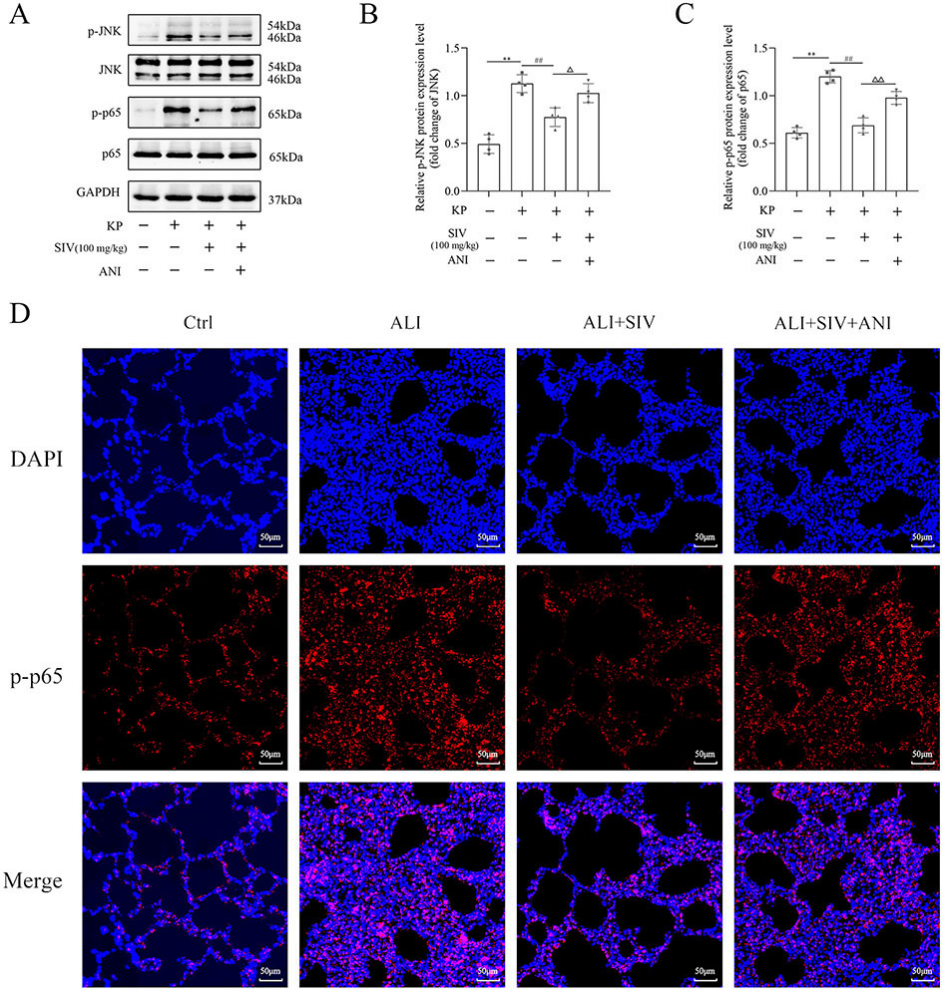
**ANI impaired SIV-suppressed activation of JNK/NF-κB signaling pathway in vivo.** Western blots showed the proteins and relative quantification levels in the (A)–(C) JNK/NF-κB signaling pathways. Immunofluorescence staining of (D) p-p65 were analyzed in different groups. *P <0.05, **P <0.01; ^#^*P* <0.05, ^##^*P* <0.01; Δ*P* <0.05, ΔΔ*P* <0.01. SIV: Sivelestat sodium; ALI: Acute lung injury; KP: *Klebsiella pneumoniae*; ANI: Anisomycin; Ctrl: Control; JNK: c-Jun N-terminal kinase; NF-κB: Nuclear factor κB.

**Figure 9.**
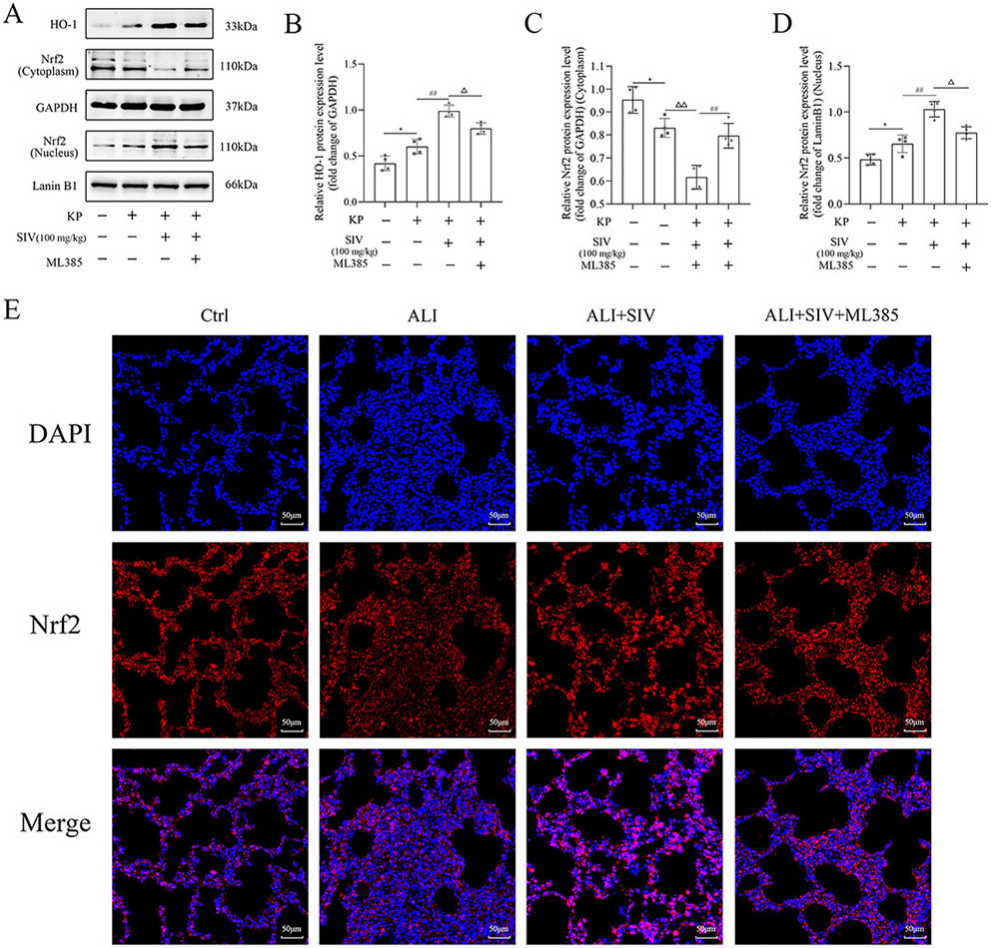
**ML385 impaired SIV-activated Nrf2/HO-1 signaling pathway in vivo.** Western blots showed the proteins and relative quantification levels in the (A)–(D) Nrf2/HO-1 signaling pathways. Immunofluorescence staining of (E) p-p65 were analyzed in different groups. *P <0.05, **P <0.01; ^#^*P* <0.05, ^##^*P* <0.01; Δ*P* <0.05, ΔΔ*P* < 0.01. SIV: Sivelestat sodium; ALI: Acute lung injury; KP: *Klebsiella pneumoniae.* Nrf2: Nuclear factor erythroid 2-related factor 2; HO-1: Heme oxygenase 1.

The oxidative stress markers in lung tissues, inflammatory factors in serum, cell count and neutrophil levels in BALF, and the Nrf2/HO-1 and JNK/NF-κB signaling pathways-related proteins’ levels were identified by immunofluorescence staining and western blotting to further explore the potential mechanisms of SIV effects in the ALI rats. The results showed that the ALI group had significantly higher MDA levels and lower GSH-Px and SOD levels, which were reversed by SIV treatment. However, ML385 inhibited the antioxidant effects of SIV (Figure 7A and 7C). Detecting the levels of the inflammatory cytokines indicated a significant increase in the IL-1β, IL-8, and TNF-α levels in the ALI group, which markedly decreased after the SIV treatment. These anti-inflammatory effects of SIV were abolished by ML385 or ANI (Figure 7D and 7F). The percentage of neutrophils and total cell count in BALF were significantly higher in the ALI groups than those in the ALI + SIV group; ML385 or ANI also inhibited these changes (Figure 7G and 7H). Western blotting revealed that the p-p65 and p-JNK levels increased in the ALI-treated rats; this effect was significantly reversed by the SIV treatment. The effects of SIV were diminished by ANI (Figure 8A and 8C). The immunofluorescence staining of p-p65 also showed that ANI inhibited the effects of SIV (Figure 8D). Moreover, there were alterations in the Nrf2/HO-1 signaling pathway-related protein levels. Western blot results indicated that SIV could promote the Nrf2’s nuclear translocation, thereby increasing the expression level of HO-1, which could be partially reversed by ML385 (Figure 9A and 9D). The immunofluorescence staining of Nrf2 indicated that SIV therapy promotes the nuclear translocation of Nrf2; however, the ML385 administration significantly inhibited the effects of SIV treatment (Figure 9E). Overall, these results suggested that SIV could alleviate oxidant damage and inflammation in ALI rats by inhibiting JNK/NF-κB and activating Nrf2/HO-1 signaling pathways.

## Discussion

SIV is mainly used to treat ALI or ARDS with systemic inflammatory response syndrome [28, 29]. However, the basic research studies on the treatment of ALI with SIV are limited. This study analyzed the oxidative stress and inflammatory cytokines markers levels to clarify the underlying effects of SIV on ALI in vivo and in vitro. This study demonstrated that the administration of SIV could markedly inhibit the JNK/NF-κB and activate the Nrf2/HO-1 signaling pathway, thereby inhibiting oxidative stress and inflammatory response.

The most common cause of ALI is microbial infections-induced severe lung infections, which are commonly caused by pathogenic bacteria and viruses. The ALI pathogenesis is characterized by severe damage of alveolar capillary endothelial cells, impaired blood–air barrier, extensive infiltration of inflammatory cells and inflammatory factors, and alveolar and interstitial lung edema, eventually leading to ARDS [30]. Lipopolysaccharide (LPS) can be used to induce ALI and is relatively simple to perform [31]. However, using bacterial infection with ALI is closer to the clinic and can better simulate the pathological changes of clinical ALI. TNF-α is an important proinflammatory factor that is released earliest in the ALI inflammatory response and can induce the release of other cytokines. Therefore, the current study used KP to induce ALI in rats in vivo and TNF-α as a single stimulus to mimic the cellular inflammatory-oxidative damage during ALI in vitro.

The main features of ALI include the extensive infiltration of immune cells and massive secretion of inflammatory cytokines and mediators, causing severe structural damage to lung tissue [32, 33]. First, this study observed that the intratracheal injection of KP in rats caused hyperplasia, solidification, alveolar luminal hemorrhage, and massive neutrophil aggregation in the lung tissues of rats as well as significant elevation of inflammatory cytokines levels in serum. These histopathological changes and levels of inflammatory cytokines were reversed by the SIV treatment, thereby reducing pulmonary edema, inflammatory response, and mortality. These results suggested that SIV had good efficacy against the KP-induced ALI. Next, the in vitro analysis indicated that TNF-α caused injury to the HPMECs, resulting in decreased cell viability and elevated levels of inflammatory cytokines; the SIV treatment could reverse these changes. This demonstrated that SIV could act directly on the HPMECs, protecting them by reducing inflammatory cytokine production. These observations were similar to the previous studies, suggesting that SIV could reduce the IL-8 levels in the TNF-α-treated A549 cells [34]. The inflammatory cytokines can not only damage the lung tissue but also attract more immune cells, including neutrophils and monocytes, into the interstitial and alveoli of the lungs, thereby further aggravating lung injury [35]. Therefore, preventing the accumulation of immune cells in lung tissue and reducing the secretion of immune cells are important measures that can mitigate lung injury. The present study showed that the SIV treatment decreased the KP-induced increase in the inflammatory cytokines levels and decreased the percentage of neutrophils and total cell count in BALF. These findings indicated that SIV could reduce the bacterial infection-induced pulmonary inflammatory response by lowering the inflammatory cytokines levels and preventing the infiltration of inflammatory cells into the lung tissue. In both in vitro and in vivo experiments, these results suggested that SIV could inhibit the inflammatory response.

The most important feature of oxidative stress is that organisms produce a large amount of ROS. When ROS exceeds the scavenging capacity of the endogenous antioxidant system, it can damage important molecular structures, such as DNA, lipids, and proteins, thereby impairing cellular functions [36, 37]. In the current study, the ROS levels were significantly elevated in the TNF-α-stimulated HPMECs in vitro. The SIV treatment significantly reduced the level of ROS. These results suggested that SIV could alleviate oxidative stress by reducing ROS production. In addition, MDA is an indicator of excessive oxidative stress and cellular injury [38]. Under normal physiological conditions, SOD can bind to superoxide anion, and GSH-Px can bind to hydrogen peroxide. These are the important endogenous antioxidant enzymes in the body, which can metabolize the excess ROS in the body [39]. In the current study, KP-induced oxidative stress, which was reflected in the significant changes in oxidative stress indicators, including increased levels of MDA and decreased levels of GSH-Px and SOD in vivo. The SIV treatment markedly inhibited the production of MDA and increased the levels of GSH-Px and SOD in the lung tissue. This showed that SIV increased the expression levels of endogenous antioxidant enzymes, thereby reducing oxidative stress. In brief, these data demonstrated that SIV might have protective effects against ALI through its anti-inflammatory and antioxidant effects in vitro and in vivo.

The JNK and NF-κB signaling pathways are critical in regulating inflammatory cytokines levels [40]. JNK pathway can be activated by an extracellular stimulus, regulating the inflammatory cytokines levels. The current study demonstrated that the TNF-α stimulated HPMECs, increasing the phosphorylation levels of JNK and NF-κB and inflammatory cytokines levels in vitro. Previous studies showed that the inhibition of the JNK phosphorylation could reduce the expression levels of inflammatory cytokines [41]. This study revealed that SIV could inhibit the phosphorylation of JNK, causing a significant decrease in inflammatory cytokines. Moreover, SIV also decreased the phosphorylation levels of NF-κB. NF-κB is present in the cytoplasm in normal conditions. However, when stimulated by an external stimulus, it is activated, followed by phosphorylation and rapid translocation into the nucleus, thereby regulating the proinflammatory factors levels [13]. Inhibition of the NF-κB activation might hinder the further development of inflammation [42]. Therefore, the current study hypothesized that SIV might inhibit the inflammatory cytokines levels by the inhibition of JNK/NF-κB signaling pathway. In order to test this hypothesis, a JNK activator (ANI) was used to observe the effects of SIV on inflammatory cytokines levels and the JNK/NF-κB signaling pathway. The results indicated that ANI decreased the effects of SIV on the inhibition of the p-JNK, thereby increasing the phosphorylation levels of NF-κB, promoting its nuclear translocation, and increasing the inflammatory cytokines levels. The effects of SIV on the JNK/ NF-κB signaling pathway were also explored in the ALI rat models. The results revealed that SIV could limit the activation of JNK and decrease the phosphorylation levels of NF-κB. However, ANI reversed these changes. Meanwhile, the data also suggested that treatment with ANI could inhibit the protective effects of SIV on ALI, such as a decrease in the elevated levels of inflammatory cytokines, total cell counts, and percentages of neutrophils in BALF, increase in the lung W/D ratio, aggravation of the lung histopathological changes, and increase in the mortality rate in rats. In addition, ANI could also exacerbate oxidative stress, which was reflected in an increase in the MDA levels and a decrease in the GSH-Px and SOD levels. This also further illustrated how oxidative stress and inflammation interacting with each other [43]. In conclusion, these data adequately demonstrated that SIV could attenuate ALI by the inhibition of JNK/NF-κB signaling pathway.

Nrf2 can regulate the synthesis of antioxidant enzymes, thereby protecting the body from oxidative damage. Nrf2/HO-1 is one of the most studied signaling pathways in the endogenous antioxidant system [44]. Increasing evidence suggests that the Nrf2/HO-1 activation might attenuate oxidative stress injury [45, 46]. The current study showed that Nrf2 was activated and followed by nuclear translocation during oxidative stress, thereby increasing the antioxidant enzyme HO-1 levels. The SIV treatment further promoted the translocation of Nrf2 into the nucleus and upregulated the levels of HO-1. However, ML385 could inhibit these changes in vitro and in vivo. Meanwhile, ML385, similar to ANI, could also inhibit the protective effects of SIV on ALI in vitro and in vivo, such as increasing the ROS levels in cells, increasing the inflammatory cytokines levels and neutrophil percentage in BALF, decreasing the SOD and GSH-Px levels in rat lung tissues, and aggravating the oxidative stress and inflammatory responses. These findings suggested that SIV could reduce ALI by activation of Nrf2/HO-1 signaling pathway.

## Conclusion

This study showed that SIV could reduce the oxidative stress and inflammatory response in the TNF-α-stimulated HPMECs and KP-induced ALI rats by the inhibition of JNK/NF-κB signaling pathway and activation of Nrf2/HO-1 signaling pathway, thereby further revealing the mechanism of the protective effects of SIV on ALI.

## Supplemental Data

**Figure S1.**
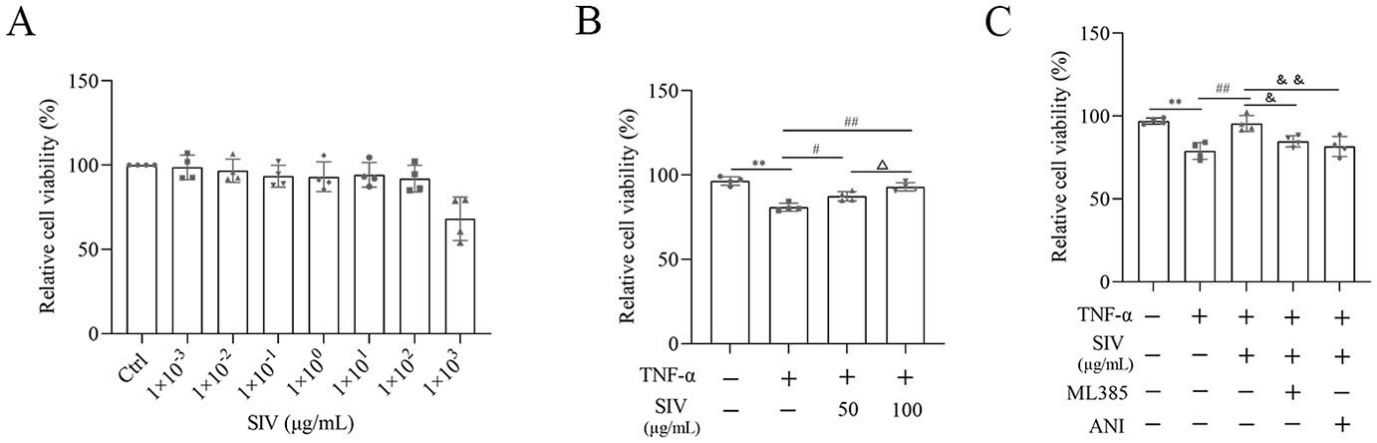
**SIV increased the viability in TNF-α-stimulated HPMECs**. (A) Viability of HPMECs treated with SIV (ranging from 0 to 1×10^3^µg/mL) for 24 h; (B) Viability of HPMECs treated with SIV (50, 100 µg/mL) and TNF-α (0.2 µg/mL) for 24 h; (C) Viability of HPMECs treated with SIV (100 µg/mL), ML385, and ANI for 24 h. *P < 0.05, **P < 0.01; ^#^*P* < 0.05, ^##^*P* < 0.01; Δ*P* < 0.05, ΔΔ*P* < 0.01; ^&^*P* < 0.05, ^&&^*P* < 0.01. SIV: Sivelestat sodium; MPMEC: Human pulmonary microvascular endothelial cells; TNF-α: Tumor necrosis factor α; ANI: Anisomycin.
